# Association of periodontal condition with insulin sensitivity – results of the Oulu1935 cohort study

**DOI:** 10.2340/aos.v85.46514

**Published:** 2026-07-16

**Authors:** Ville Myllymäki, Pekka Ylöstalo, Anna Liisa Suominen, Matti Knuuttila, Ulla Rajala, Sirkka Keinänen-Kiukaanniemi, Sirpa Anttila, Tuomas Saxlin

**Affiliations:** a Department of Oral and Maxillofacial Diseases, University of Helsinki and Helsinki University Hospital, Helsinki, Finland; b Institute of Dentistry, University of Eastern Finland, Kuopio, Finland; c Medical Research Center Oulu, Oulu University Hospital and University of Oulu, Oulu, Finland; d Department of Oral and Maxillofacial Surgery, Oulu University Hospital, Oulu, Finland; e Research Unit of Population Health, University of Oulu, Oulu, Finland; f Oral and Maxillofacial Diseases Teaching Unit, Wellbeing Services County of Northern Savo, Kuopio, Finland; g Welfare Epidemiology and Monitoring Unit, Finnish Institute for Health and Welfare, Helsinki, Finland; h Unit of Primary Health Care, Oulu University Hospital, Oulu, Finland; i Wellbeing Services County of North Ostrobothnia, Pyhäjärvi, Finland; j Dental Training Clinic, Oral Health Care, Wellbeing Services County of North Ostrobothnia, Oulu, Finland

**Keywords:** Epidemiology, insulin resistance, periodontal diseases, longitudinal studies, oral health

## Abstract

**Objective:**

To investigate whether baseline periodontal condition predicts reduced insulin sensitivity (RIS) over a follow-up period of about 15 years among participants with baseline euglycemia.

**Material and methods:**

A subpopulation (*n* = 224) of the Oulu1935 cohort was studied. Periodontal condition was defined by the presence and number of sites with periodontal pockets (probing pocket depth ≥ 4 mm) or by the participant being edentulous. Insulin sensitivity was measured using the Quantitative Insulin Sensitivity Check Index, with the lowest tertile indicating RIS. Rate ratios (RRs) with 95% confidence intervals (CI) were estimated using Poisson regression models with a robust error variance.

**Results:**

RIS was observed in 21.3% of the dentate participants without periodontal pockets, 32.8% of those with 1–6 sites with pockets, 33.3% of those with ≥ 7 sites with pockets, and 40.8% of the edentulous participants. Furthermore, adjusted RRs with 95% CIs (in parentheses) for the association of periodontal condition with RIS (reference: dentate participants without periodontal pockets) were 1.4 (0.8–2.6), 1.1 (0.6–2.1), and 1.4 (0.8–2.7), respectively.

**Conclusions:**

Poor periodontal condition and edentulousness predicted RIS. A healthy periodontium appears to support normal insulin function, but further studies are needed.

## Introduction

Insulin is a peptide hormone that plays a vital role in energy metabolism, particularly in maintaining glucose homeostasis, whereas insulin resistance (IR) is characterized by a reduced responsiveness of insulin target tissues to physiological levels of insulin. Several methods exist to assess reduced insulin sensitivity (RIS) and IR, such as the Quantitative Insulin Sensitivity Check Index (QUICKI) and the Homeostatic Model Assessment for Insulin Resistance. The well-known risks for IR include being overweight or obese, low physical activity, increased waist and neck circumferences, as well as genetic predisposition [1].

As IR itself is a metabolic entity and regarded as a pathophysiological condition, it is also an essential component, along with impaired insulin secretion, in the development of both prediabetes (impaired fasting glucose and/or impaired glucose tolerance) and subsequent diabetes mellitus (DM) [1]. These conditions have emerged as serious global health threats in recent decades. In 2024, it was estimated that nearly one-third of adults aged 20–79 years (1,700 million individuals) had prediabetes or DM, with projections indicating an increase in the prevalence to over one-third (2,300 million individuals) by 2050 [2]. This highlights the need to utilize all available methods to prevent known risk factors for glycemic disorders and to explore new ones.

Periodontitis, an inflammatory disease of the tooth-supporting structures, has also recently been a target of interest associated with IR. Like glycemic disorders, periodontitis is also a prevalent disease globally. The global prevalence of severe periodontitis was reported to be slightly more than 12% (1,100 million individuals) in 2021, and the situation is predicted to worsen; the prevalence is expected to exceed 13% (1,500 million individuals) by 2050 [3–4]. The suggested main mechanisms explaining the association of periodontitis with IR include low-level systemic inflammation due to the release of pro-inflammatory cytokines and bacterial products into the bloodstream from inflamed periodontal tissues [5].

The association of periodontitis with IR has been examined in several studies, but there have been substantial differences in the study design, study populations, and the assessment methodology, which make inferences difficult. Many cross-sectional studies have shown that periodontitis is associated with RIS [6–9], but somewhat contradictory findings also exist [10–11]. However, longitudinal studies on the association of periodontitis with RIS or IR are, to the best of our knowledge, non-existent.

To address the scarcity of longitudinal evidence on whether poor periodontal condition is associated with RIS, this longitudinal cohort study investigated whether baseline periodontal condition – defined by the presence and number of sites with periodontal pockets (probing pocket depth [PPD] ≥ 4 mm) or by edentulousness – predicts RIS over a follow-up period of about 15 years among individuals with baseline euglycemia (i.e. neither prediabetic nor diabetic). The hypothesis was that those with poor periodontal condition at baseline are more likely to have RIS at follow-up.

## Material and methods

**Figure 1:**
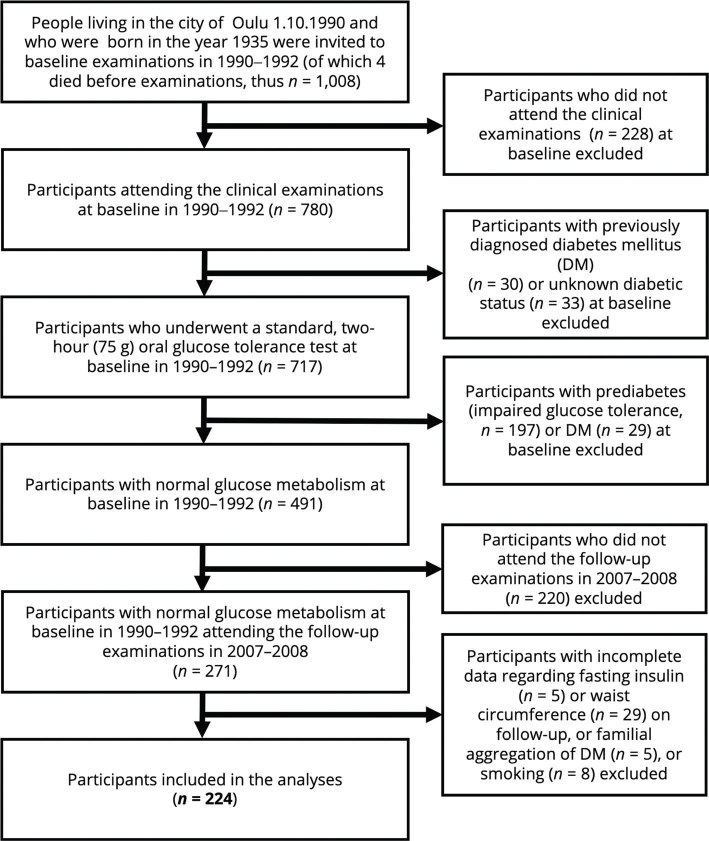
Schematic representation of the study population utilized in this study.

The Oulu1935 cohort study was a study on an aging population in Oulu, a medium-sized city in northern Finland. A total of 1,008 individuals, who were born in 1935 and lived in the city of Oulu on 1.10.1990, were invited to the baseline examinations in 1990–1992, in which 780 eventually participated. The follow-up examinations were performed in 1996–1998, 2007–2008, and 2015–2016. In the present study, data from the baseline examinations and the follow-up examinations in 2007–2008 were primarily used; data from the follow-up examinations in 1996–1998 were used as compensatory data in case measurements for some variables were not performed at baseline in 1990–1992. The data were collected through structured questionnaires, interviews, clinical examinations, and laboratory tests. The exclusion criteria for the present study were nonparticipation in the clinical examinations and laboratory tests at baseline or at follow-up, previously diagnosed DM at baseline, and baseline impaired glucose tolerance (2‐hour post‐load blood glucose value 7.8–11.1 mmol/L) or DM (2‐hour post‐load blood glucose value ≥ 11.1 mmol/L) based on a standard 2-hour (75 g) oral glucose tolerance test and the contemporary guidelines defined by the World Health Organization (WHO) [12]. Those with incomplete data on diabetic status at baseline, fasting blood insulin level, or waist circumference (WC) at follow-up, the familial aggregation of DM, or smoking were also excluded from the study. Thus, the present study utilized a subpopulation of 224 participants of the Oulu1935 cohort study. A detailed description of the formation of the study population is presented in Figure 1.

### Outcome

The outcome variable in this study was RIS in the follow-up examinations in 2007–2008. Insulin sensitivity (IS) was assessed using QUICKI, calculated as 1/[log(I0) + log(G0)], where I0 and G0 denote fasting insulin and glucose, respectively. Serum immunoreactive insulin levels were determined by radioimmunoassay using the Phadeseph Insulin RIA100 kit (Pharmacia Diagnostics AB, Uppsala, Sweden), which is highly sensitive to proinsulin and its conversion products. The assay has a proinsulin cross-reactivity of about 41% [13]. Furthermore, the blood glucose levels were measured by the hexokinase-glucose-6-phosphate dehydrogenase method [14]. The occurrence of RIS at follow-up was treated as a dichotomous variable (yes/no) in the statistical analyses and was defined based on the QUICKI value in the lowest tertile of the study population (≤ 0.325).

### Exposure

The exposure variable was baseline periodontal condition, defined by the presence and number of sites with periodontal pockets (PPD ≥ 4 mm) or whether the participant was edentulous (neither teeth nor dental implants) at baseline. Periodontal examinations were performed as a part of the clinical oral examinations at baseline between 1990 and 1991. PPDs were measured from four sites (mesial, distal, buccal, and palatal/lingual) of each tooth (except radices), and measurements ≥ 4 mm were registered. Two examiners, who received identical instructions, conducted the measurements. The intra-examiner agreement in the measurements of deepened periodontal pockets was 96.7% and the kappa statistic 0.80 for examiner 1, and 95.3% and 0.78 for examiner 2. For the statistical analyses, participants were grouped into four categories: dentate participants without periodontal pockets, those with 1–6 sites with periodontal pockets, those with ≥ 7 sites with periodontal pockets, and edentulous participants. The categorization threshold was determined based on the median number of sites exhibiting PPD ≥ 4 mm.

### Covariates

Potential confounders were sex, educational level, annual household income, familial aggregation of DM, physical activity, food consumption habits, smoking, WC, change in WC during the follow-up (between the follow-up examinations in 1996–1998 and 2007–2008), serum triglyceride and high-density lipoprotein cholesterol (HDL-C) levels, and arterial hypertension.

Participants’ educational level was assessed using a postal questionnaire at baseline and was divided into three categories for statistical analyses: basic (vocational school or lower), intermediate (graduated from a vocational college or university of applied sciences), and higher (graduated from a university), based on the international standard classification of education [15]. The data on annual household income were also gathered based on the baseline postal questionnaire and were grouped into four categories: < 20,400€, 20,400–30,599€, 30,600–40,800€, and > 40,800€ per year.

The familial aggregation of DM was determined based on whether the participants had close family members with the condition. This information was gathered in an interview during the follow-up examinations in 2007–2008, in which the participants were asked: ‘Has anyone in your immediate family or any relatives been diagnosed with diabetes mellitus?’ The three categorical response options presented to the participant were ‘No’, ‘Yes, diabetes has been diagnosed in grandparents, aunts, uncles, or cousins (but not in parents, siblings, or children)’, or ‘Yes, diabetes has been diagnosed in parents, siblings, or children’, with the first option depicting a low hereditary risk, the middle option depicting a moderate hereditary risk, and the last option depicting a high hereditary risk.

Information about physical activity was obtained using the baseline postal questionnaire. The participants were asked: ‘How many minutes do you walk or cycle daily on your way to work?’ and ‘How many times a week do you exercise for at least half an hour in your leisure time?’ The response options for the former question were: ‘I don’t work or I work from home’, ‘I travel to work using motorized vehicles’, ‘Less than 15 minutes’, ‘15–30 minutes’, ‘30–60 minutes’, and ‘More than one hour’. For the latter question, the response options were: ‘Never’. ‘Less than once a month’, ‘1–2 times a month’, ‘Once a week’, ‘2–3 times a week’, and ‘5–7 times a week’. Physical activity was classified as low if the participant walked or cycled for less than 15 minutes on their way to work and exercised only once a week or less in their leisure time. If participants exceeded these levels of activity, their physical activity was categorized as high.

Food consumption habits were assessed based on three key questions in the baseline postal questionnaire. Participants were asked: ‘How often do you eat vegetables, fruits, or root crops?’ with the response options being ‘Daily’ or ‘Less than once a week’. They were also asked: ‘What type of fat do you use on bread?’ with the response options being ‘Nothing or light margarine’ or ‘Butter’. Lastly, they were asked: ‘What is the fat content of the drink you have with meals?’ with the response options being ‘Non-fat (e.g., water, or fat-free milk or sour milk)’, ‘Low-fat (e.g., light milk or sour milk)’, or ‘High-fat (e.g., fatty milk)’. Based on their answers, participants were categorized into three dietary groups. Those who selected the healthiest responses for all three questions were classified as having a healthy diet. If they chose one unhealthy option, they were categorized as having a moderately healthy diet. Participants who chose more than one unhealthy option were classified as having an unhealthy diet.

Information about smoking habits was collected via a questionnaire completed at follow-up in 2007–2008, in which participants were asked whether they had ever smoked regularly, defined as smoking almost every day for at least 1 year in their life. They could answer ‘Yes’ or ‘No’. They were also asked about their current smoking status, with response options being ‘Regularly’, ‘Occasionally’, or ‘No smoking’. If they had smoked in the past, they were asked at what age they quit smoking. Based on the answers, participants were classified into three groups: never smokers, former smokers, and current smokers. Those who answered ‘No’ to having ever smoked regularly were categorized as never smokers. Participants were classified as former smokers if they had answered the question about when they quit smoking. Current smokers were those who reported smoking regularly at the time of the study. For the regression analyses, never-smokers and former smokers were combined into a single ‘non-smoker’ category.

WC as an indicator of overweight and obesity was measured in a horizontal plane, halfway between the lowest rib and iliac crest, by a trained research nurse in the follow-up examinations in 1996–1998 and 2007–2008. WC at follow-up in 2007–2008 and the change in WC during the follow-up (between the follow-up examinations in 1996–1998 and 2007–2008) were used as variables in the statistical analyses. WC at follow-up was categorized according to the WHO [16] criteria as follows: normal, ≤ 94 cm for men and ≤ 80 cm for women; increased risk of metabolic complications, >94–102 cm for men and > 80–88 cm for women; and substantially increased risk of metabolic complications, > 102 cm for men and > 88 cm for women. Furthermore, the change in WC during the follow-up was treated as a continuous variable in the regression analyses.

Serum triglyceride and HDL-C levels were measured during the follow-up examinations in 2007–2008 using venous blood samples taken after a 10–12-hour fast. The triglycerides and HDL-C were analyzed using a manual enzymatic CHOD-PAP method (Boehringer Mannheim, Mannheim, Germany) [17]. For HDL-C measurement, low-density and very-low-density lipoproteins were first precipitated with a reagent containing phosphotungstic acid and magnesium chloride. After centrifugation, the HDL-C remaining in the supernatant was measured using the CHOD-PAP method. The serum triglyceride and HDL-C levels at follow-up were used as continuous variables in the statistical analyses. Furthermore, information on arterial hypertension, diagnosed by a physician, was collected through a questionnaire during the follow-up examinations in 2007–2008 and was categorized as yes vs no.

### Ethical considerations

Ethical approval for the study was obtained from the Ethics Committee of the Faculty of Medicine of the University of Oulu, Oulu, Finland, and all the participants provided written informed consent. This study was conducted according to the Declaration of Helsinki and the guidelines developed by the Strengthening the Reporting of Observational Studies in Epidemiology (STROBE) initiative.

### Statistical analyses

**Figure 2:**
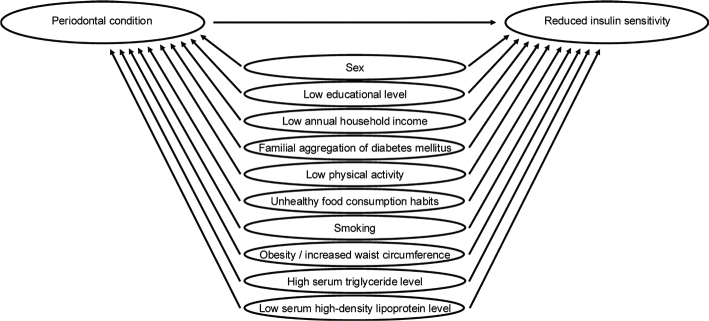
Model depicting the association between periodontal condition at baseline, covariates, and the presence of reduced insulin sensitivity at follow‐up.

Poisson regression models with robust error variance [18] were used to estimate the rate ratios (RRs) with their 95% confidence intervals (CIs). The covariates were selected based on *a priori* knowledge of their role as risk factors for RIS. Missing data were handled by exclusions or by creating an extra category ‘missing data’ for the missing observations in categorized variables. Moreover, arterial hypertension was excluded from the final models due to a high proportion of missing data and to avoid overparameterization (Figure 2). This exclusion had a negligible impact on the estimates. Furthermore, regression analyses were performed for the total study population and restricted to the non-smoking participants, to the non-smoking participants without substantially increased risk of metabolic complications, and to the non-smoking participants without substantially increased risk of metabolic complications with ≥ 5 missing teeth. These sensitivity analyses were performed to further handle confounding related to smoking [19], excessive body weight, and tooth loss. All statistical analyses were performed using SPSS software (version 29.0.2.0; SPSS, Chicago, IL, USA).

## Results

The general characteristics of the study population (*n* = 224), according to baseline periodontal condition and the occurrence of RIS at follow-up, are presented in Tables 1 and 2, respectively. At follow-up, RIS was observed in 21.3% of dentate participants without periodontal pockets, 32.8% of those with 1–6 sites with periodontal pockets, 33.3% of those with ≥ 7 sites with periodontal pockets, and 40.8% of the edentulous participants.

**Table 1:** General characteristics of the study population (*n* = 224) according to the categories of periodontal condition at baseline in 1990–1992.

	Periodontal condition				
	Dentate without periodontal pockets (probing pocket depths [PPDs] < 4 mm)	Dentate with 1–6 sites with periodontal pockets (PPD ≥ 4 mm)	Dentate with ≥ 7 sites with periodontal pockets	Edentulous	Total
Sex (baseline data), *n* (%)					
Men	21 (45)	21 (36)	26 (54)	17 (24)	85 (38)
Women	26 (55)	37 (64)	22 (46)	54 (76)	139 (62)
Number of teeth (baseline data), mean (SD)	15.0 (8.5)	17.4 (8.1)	19.8 (7.2)	-	17.4 (8.2)^†^
Reduced insulin sensitivity^‡^ (follow-up data), *n* (%)					
Yes	10 (21)	19 (33)	16 (33)	29 (41)	74 (33)
No	37 (79)	39 (67)	32 (67)	42 (59)	150 (67)
Educational level (baseline data), *n* (%)					
Basic	37 (78)	45 (78)	34 (71)	68 (96)	184 (82)
Intermediate	5 (11)	6 (10)	10 (21)	2 (3)	23 (10)
High	5 (11)	7 (12)	4 (8)	1 (1)	17 (8)
Missing data	0 (0)	0 (0)	0 (0)	0 (0)	0 (0)
Annual household income (baseline data), *n* (%)					
< €20,400	10 (21)	10 (17)	9 (19)	27 (38)	56 (25)
€20,400–30,599	10 (21)	19 (33)	6 (13)	25 (35)	60 (27)
€30,600–40,800	12 (26)	15 (26)	16 (33)	12 (17)	55 (25)
> €40,800	12 (26)	11 (19)	15 (31)	5 (7)	43 (19)
Missing data	3 (6)	3 (5)	2 (4)	2 (3)	10 (4)
Familial aggregation of diabetes mellitus (follow-up data), *n* (%)					
Low risk	24 (51)	32 (55)	33 (69)	42 (59)	131 (59)
Increased risk	23 (49)	26 (45)	15 (31)	29 (41)	93 (41)
Physical activity (baseline data), *n* (%)					
High physical activity	37 (79)	47 (81)	31 (65)	55 (78)	170 (76)
Low physical activity	10 (21)	9 (16)	16 (33)	15 (21)	50 (22)
Missing data	0 (0)	2 (3)	1 (2)	1 (1)	4 (2)
Food consumption habits (baseline data), *n* (%)					
Healthy diet	29 (62)	37 (64)	22 (46)	29 (1)	117 (52)
Moderately healthy diet	8 (17)	13 (23)	15 (31)	22 (31)	58 (26)
Unhealthy diet	6 (13)	6 (10)	9 (19)	15 (21)	36 (16)
Missing data	4 (8)	2 (3)	2 (4)	5 (7)	13 (6)
Smoking (follow-up data), *n* (%)					
Never smoker	25 (53)	34 (59)	12 (25)	25 (35)	96 (43)
Former smoker	19 (41)	22 (38)	29 (60)	38 (54)	108 (48)
Smoker	3 (6)	2 (3)	7 (15)	8 (11)	20 (9)
Waist circumference (WC [cm], [follow-up data]), mean (SD)	87.8 (7.1) (M)	93.6 (7.2) (M)	97.0 (10.0) (M)	104.5 (19.5) (M)	95.4 (12.6) (M)
	83.4 (10.1) (W)	81.0 (10.9) (W)	85.1 (12.4) (W)	86.4 (11.7) (W)	84.2 (11.4) (W)
WC^§^ (follow-up data), *n* (%)					
Normal	25 (53)	31 (54)	23 (48)	23 (32)	102 (45)
IRMC	15 (32)	17 (29)	8 (17)	20 (28)	60 (27)
SIRMC	7 (15)	10 (17)	17 (35)	28 (40)	62 (28)
WC change (cm) during the follow-up period^¶^, mean (SD)	-0.8 (5.7)	1.1 (5.9)	1.3 (7.4)	1.9 (7.9)	1.0 (6.9)
WC change (cm) during the follow-up period^¶^, *n* (%)					
WC change negative	28 (59)	28 (48)	22 (46)	28 (39)	106 (47)
0 ≤ WC change < 2	5 (11)	4 (7)	6 (12)	6 (9)	21 (9)
2 ≤ WC change < 4	5 (11)	10 (17)	2 (4)	9 (13)	26 (12)
WC change ≥ 4	9 (19)	16 (28)	18 (38)	28 (39)	71 (32)
Serum triglyceride level (follow-up data), mean (SD)	1.1 (0.3)	1.2 (0.5)	1.2 (0.5)	1.3 (0.6)	1.2 (0.6)
Serum high-density lipoprotein cholesterol level (follow-up data), mean (SD)	1.7 (0.5)	2.0 (0.5)	1.7 (0.4)	1.8 (0.5)	1.7 (0.5)
Arterial hypertension (follow-up data), *n* (%)					
No	18 (38)	29 (50)	20 (42)	28 (39)	95 (42)
Yes	18 (38)	22 (38)	23 (48)	29 (41)	92 (41)
Missing data	11 (24)	7 (12)	5 (10)	14 (20)	37 (17)

SD: standard deviation.

† Among dentate participants.

‡ Defined based on the Quantitative Insulin Sensitivity Check Index (QUICKI) value in the lowest tertile of the study population (≤ 0.325). QUICKI was calculated according to the following equation: 1/[log(I0) + log(G0)], where I0 represents fasting insulin and G0 represents fasting glucose, both measured during the clinical examination.

§ The cut-off points for waist circumference: normal, ≤ 94 cm for men and ≤ 80 cm for women; increased risk of metabolic complications (IRMC), > 94–102 cm for men and > 80–88 cm for women; and substantially increased risk of metabolic complications (SIRMC), > 102 cm for men and > 88 cm for women.

¶ Between the follow-up examinations in 1996–1998 and 2007–2008.

**Table 2:** General characteristics of the study population (*n* = 224) according to the presence of reduced insulin sensitivity (RIS)^†^ at follow-up in 2007–2008.

	RIS^†^		
	Yes	No	Total
Sex (baseline data), *n* (%)			
Men	34 (46)	51 (34)	85 (38)
Women	40 (54)	99 (66)	139 (62)
Periodontal condition (baseline data), *n* (%)			
Dentate without periodontal pockets (probing pocket depths [PPDs] < 4 mm)	10 (13)	37 (25)	47 (21)
Dentate with 1–6 sites with periodontal pockets (PPD ≥ 4 mm)	19 (26)	39 (26)	58 (26)
Dentate with ≥ 7 sites with periodontal pockets	16 (22)	32 (21)	48 (21)
Edentulous	29 (39)	42 (28)	71 (32)
Educational level (baseline data), *n* (%)			
Basic	64 (87)	120 (80)	184 (82)
Intermediate	4 (5)	19 (13)	23 (10)
High	6 (8)	11 (7)	17 (8)
Missing data	0 (0)	0 (0)	0 (0)
Annual household income (baseline data), *n* (%)			
< €20,400	16 (22)	40 (27)	56 (25)
€20,400–30,599	19 (26)	41 (27)	60 (27)
€30,600–40,800	20 (27)	35 (23)	55 (25)
> €40,800	18 (24)	25 (17)	43 (19)
Missing data	1 (1)	9 (6)	10 (4)
Familial aggregation of diabetes mellitus (follow-up data), *n* (%)			
Low risk	41 (55)	90 (60)	131 (59)
Increased risk	33 (45)	60 (40)	93 (41)
Physical activity (baseline data), *n* (%)			
High physical activity	115 (77)	55 (74)	170 (76)
Low physical activity	33 (22)	17 (23)	50 (22)
Missing data	2 (1)	2 (3)	4 (2)
Food consumption habits (baseline data), *n* (%)			
Healthy diet	32 (43)	85 (57)	117 (52)
Moderately healthy diet	21 (28)	37 (25)	58 (26)
Unhealthy diet	16 (22)	20 (13)	36 (16)
Missing data	5 (7)	8 (5)	13 (6)
Smoking (follow-up data), *n* (%)			
Never smoker	32 (43)	64 (43)	96 (43)
Former smoker	37 (50)	71 (47)	108 (48)
Smoker	5 (7)	15 (10)	20 (9)
Waist circumference (WC [cm], [follow-up data]), mean (SD)	102.1 (15.4) (M)	90.9 (7.8) (M)	95.4 (12.6) (M)
	92.4 (12.0) (W)	80.9 (9.4) (W)	84.2 (11.4) (W)
WC^‡^ (follow-up data), *n* (%)			
Normal	15 (20)	87 (58)	102 (45)
IRMC	23 (31)	37 (25)	60 (27)
SIRMC	36 (49)	26 (17)	62 (28)
WC change (cm) during the follow-up period^§^, mean (SD)	2.8 (8.2)	0.1 (6.0)	1.0 (6.9)
WC change (cm) during the follow-up period^§^, *n* (%)			
WC change negative	29 (39)	77 (51)	106 (47)
0 ≤ WC change < 2	3 (4)	18 (12)	21 (9)
2 ≤ WC change < 4	10 (14)	16 (11)	26 (12)
WC change ≥ 4	32 (43)	39 (26)	71 (32)
Serum triglyceride level (follow-up data), mean (SD)	1.5 (0.7)	1.1 (0.5)	1.2 (0.6)
Serum high-density lipoprotein cholesterol level (follow-up data), mean (SD)	1.5 (0.4)	1.8 (0.5)	1.8 (0.5)
Arterial hypertension (follow-up data), *n* (%)			
No	27 (36)	68 (46)	95 (42)
Yes	39 (53)	53 (35)	92 (41)
Missing data	8 (11)	29 (19)	37 (17)

SD: standard deviation.

† Defined based on the Quantitative Insulin Sensitivity Check Index (QUICKI) value in the lowest tertile of the study population (≤ 0.325). QUICKI was calculated according to the following equation: 1/[log(I0) + log(G0)], where I0 represents fasting insulin and G0 represents fasting glucose, both measured during the clinical examination.

‡ The cut-off points for waist circumference: normal, ≤ 94 cm for men and ≤ 80 cm for women; increased risk of metabolic complications (IRMC), > 94–102 cm for men and > 80–88 cm for women; and substantially increased risk of metabolic complications (SIRMC), > 102 cm for men and >88 cm for women.

§ Between the follow-up examinations in 1996–1998 and 2007–2008.

The results of the unadjusted and adjusted regression analyses in the total study population are presented in Table 3. In the regression analyses adjusted for confounding factors, dentate participants with periodontal pockets or those who were edentulous at baseline had a higher likelihood of having RIS at follow-up than dentate participants without periodontal pockets at baseline. The adjusted RRs with 95% CIs (in parentheses) for the dentate participants with 1–6 sites with periodontal pockets, for those with ≥ 7 sites with periodontal pockets, and for the edentulous participants were 1.4 (0.8–2.6), 1.1 (0.6–2.1), and 1.4 (0.8–2.7), respectively. However, the results of the regression analyses in the total study population failed to reach statistical significance.

**Table 3:** Association of periodontal condition at baseline in 1990–1992 with the presence of reduced insulin sensitivity (RIS)^†^ at follow-up in 2007–2008; unadjusted and adjusted^‡^ rate ratios (RR) with 95% confidence intervals (95% CI) in the total study population (effective *n* = 224).

Periodontal condition	RIS^†^	
	Unadjusted RR (95% CI)	Adjusted^‡^ RR (95% CI)
Dentate without periodontal pockets (probing pocket depths [PPDs] < 4 mm)	1	1
Dentate with periodontal pockets (PPD ≥ 4 mm)	1.6 (0.8–2.9)	1.3 (0.8–2.7)
1–6 sites with periodontal pockets	1.5 (0.8–3.0)	1.4 (0.8–2.6)
≥ 7 sites with periodontal pockets	1.6 (0.8–3.1)	1.1 (0.6–2.1)
Edentulous	1.9 (1.0–3.6)	1.4 (0.8–2.7)

† Defined based on the Quantitative Insulin Sensitivity Check Index (QUICKI) value in the lowest tertile of the study population (≤ 0.325). QUICKI was calculated according to the following equation: 1/[log(I0) + log(G0)], where I0 represents fasting insulin and G0 represents fasting glucose, both measured during the clinical examinations.

‡ Adjusted for sex, educational level, annual household income at baseline, familial aggregation of diabetes mellitus, physical activity at baseline, food consumption habits at baseline, smoking at follow-up, waist circumference (WC) at follow-up, change in WC during the follow-up period (between the follow-up examinations in 1996–1998 and 2007–2008) (continuous variable), and serum triglyceride and high-density lipoprotein cholesterol levels at follow-up (continuous variables).

The results of the sensitivity analyses restricted to the non-smoking participants, the non-smoking participants without substantially increased risk of metabolic complications, and the non-smoking participants without substantially increased risk of metabolic complications with ≥ 5 missing teeth are presented in Table 4. The association mainly followed a similar pattern, although it was in many cases stronger, as in the analyses among the total study population.

**Table 4:** Association of periodontal condition at baseline in 1990–1992 with the presence of reduced insulin sensitivity (RIS)^†^ at follow-up in 2007–2008; analyses restricted to non-smoking^‡^ participants, to non-smoking participants without substantially increased risk of metabolic complications (SIRMC)^§^, and to non-smoking participants without SIRMC with ≥ 5 missing teeth^¶^. Unadjusted and adjusted^††^ rate ratios (RR) with 95% confidence intervals (95% CI).

Periodontal condition	RIS^†^		
	Unadjusted RR (95% CI); non-smokers	Unadjusted RR (95% CI); non-smokers without SIRMC	Unadjusted RR (95% CI); non-smokers without SIRMC with ≥ 5 missing teeth
Dentate without periodontal pockets (probing pocket depths [PPDs] < 4 mm)	1	1	1
Dentate with periodontal pockets (PPD ≥ 4 mm)	1.7 (0.9–3.2)	1.5 (0.7–3.6)	1.4 (0.6–3.2)
1–6 sites with periodontal pockets	1.7 (0.8–3.3)	2.0 (0.8–4.6)	1.8 (0.8–4.1)
≥ 7 sites with periodontal pockets	1.7 (0.8–3.4)	0.7 (0.2–2.7)	0.7 (0.2–2.5)
Edentulous	2.1 (1.1–4.0)	1.7 (0.7–4.2)	1.5 (0.6–3.7)

**Periodontal condition**	**RIS** ^†^		

	**Adjusted**^††^ **RR (95% CI); non-smokers**	**Adjusted**^††^ **RR (95% CI); non-smokers without SIRMC**	**Adjusted**^††^ **RR (95% CI); non-smokers without SIRMC with ≥ 5 missing teeth**

Dentate without periodontal pockets	1	1	1
Dentate with periodontal pockets	1.4 (0.8–2.6)	1.8 (0.8–3.9)	1.7 (0.8–3.7)
1–6 sites with periodontal pockets	1.7 (0.9–3.0)	2.5 (1.1–5.5)	2.3 (1.0–5.3)
≥ 7 sites with periodontal pockets	1.2 (0.6–2.3)	0.9 (0.3–3.3)	0.9 (0.2–3.3)
Edentulous	1.5 (0.8–2.9)	1.6 (0.6–4.2)	1.5 (0.6–3.9)

† Defined based on the Quantitative Insulin Sensitivity Check Index (QUICKI) value in the lowest tertile of the study population (≤ 0.325). QUICKI was calculated according to the following equation: 1/[log(I0) + log(G0)], where I0 represents fasting insulin and G0 represents fasting glucose, both measured during the clinical examinations.

‡ Current smokers excluded. Effective *n* = 204.

§ Current smokers and participants with SIRMC (waist circumference [WC] > 102 cm for men and > 88 cm for women at follow-up) excluded. Effective *n* = 145.

¶ Current smokers and participants with SIRMC and < 5 missing teeth excluded. Effective *n* = 137.

†† Adjusted for sex, educational level, annual household income at baseline, familial aggregation of diabetes mellitus, physical activity at baseline, food consumption habits at baseline, WC at follow-up, change in WC during the follow-up period (between the follow-up examinations in 1996–1998 and 2007–2008) (continuous variable), and serum triglyceride and high-density lipoprotein cholesterol levels at follow-up (continuous variables).

## Discussion

In this population of euglycemic (i.e. neither prediabetic nor diabetic) participants at baseline, poor periodontal condition and edentulousness appeared to predict RIS. In fact, at follow-up, the proportion of the participants with RIS was about 50% higher for participants with periodontal pockets and almost 100% higher for edentulous participants than for dentate participants without periodontal pockets.

Besides predicting RIS, poor periodontal condition and edentulousness were also associated with increased risk for it after adjusting for confounding factors. However, this association appeared to be weak, inconsistent, and in most cases statistically non-significant. The association is also confounded by other factors, as it attenuated after adjusting for other risk factors, and strengthened in sensitivity analyses carried out in a homogenous subpopulation, i.e. participants who did not smoke and further to those who had a lower risk of metabolic complications and at least five missing teeth.

The strengths of this study include the longitudinal study design with a long follow-up period, which allowed the determination of a temporal sequence in the association between periodontal condition and RIS. However, a lengthy follow-up period may result in loss to follow-up, potentially introducing attrition bias, i.e. leading to either an underestimation or overestimation of the observed association. Nonetheless, typically in longitudinal studies involving the elderly, people who are ill or frail are more likely to drop out. Therefore, it can be reasonably assumed that the possible attrition bias in this study may have caused underestimation of the strength of the association of periodontal condition with RIS. Still, the potential effect of loss to follow-up should be considered when assessing the generalizability of the results.

An additional strength of this study, beyond its lengthy follow-up, is that it accounted for several risk factors for RIS and restricted sensitivity analyses to specific subpopulations – i.e. non-smokers, non-smokers without substantially increased risk of metabolic complications, and non-smokers without substantially increased risk of metabolic complications with ≥ 5 missing teeth – in order to minimize the potential bias related to confounding. However, it must be acknowledged that residual confounding may exist, since all the risk factors for RIS may not yet have been identified, and consequently could not been controlled for. Residual confounding may also exist due to unfit operationalization of the known risk factors, especially smoking, physical activity, and eating behaviors. In addition, a drawback of the restrictions is the diminished statistical power of the analyses.

The exposure in this study, the number of sites with deepened periodontal pockets, is a commonly utilized approach in both clinical practice and research for the assessment of periodontal tissue health. Furthermore, PPDs are a reliable indicator of the current inflammatory condition of the periodontium [20], and the relevance of deepened periodontal pockets is corroborated by Pérez‑Chaparro et al. [21], who reported substantial microbial differences between shallow and moderate‑to‑deep PPD sites. However, the lack of information about additional periodontal parameters, such as gingival bleeding, exact site-specific PPDs, or attachment loss, thereby precluding a more detailed classification of periodontal tissue health and disease, can be considered a limitation. Moreover, the assessment of periodontal condition only at baseline can also be considered a limitation, since the evolution of disease activity throughout the follow-up period could not be taken into consideration. However, the relevance of deepened periodontal pockets is corroborated by a subpopulation of the present study had periodontal re-examination done in connection with the health examinations of the 1996–1998 follow-up, and, on average, no notable change was observed in the number of sites with periodontal pockets during this period [22]. The view that periodontal tissue destruction typically progresses slowly – often over many years or even decades – is further reinforced by findings from Eke et al. [23], based on a nationally representative sample of U.S. adults aged 30 years and older. Their results indicated that the age‑related rise in overall periodontitis prevalence was largely attributable to an increase in moderate disease, whereas the prevalences of mild and severe periodontitis showed only modest increases.

In addition, active periodontal treatment or supportive periodontal therapy over the course of the follow-up period could not be accounted for. Nevertheless, several studies have reported that non-surgical periodontal treatment [24] or a combination of surgical and non-surgical periodontal treatment [25] and also local antimicrobial periodontal treatment [26] can decrease IR. It is therefore reasonable to assume that the possible periodontal treatment during the follow-up period could have also had a favorable effect on the IS of these participants and may have thus led to underestimation of the magnitude of the association detected. Furthermore, missing information about anti-inflammatory medication and the timing and reason for tooth loss, as well as the use of dentures, can also be considered a limitation of the present study.

The golden standard for measuring IS in humans is the euglycemic hyperinsulinemic clamp; however, its complexity has led to the creation and validation of numerous surrogate indices, such as QUICKI [27]. It is simple, reliable, reproducible, and has excellent positive predictive power for determining the development of DM [28]. It is also inexpensive and minimally invasive, making it suitable for use in epidemiological studies [29]. In comparison with other surrogate indices of IS, QUICKI is the most thoroughly evaluated and validated [30]. Nevertheless, although it has several merits as a measure of IS, the lack of a universally accepted threshold for RIS can be considered a potential threat to validity. Creating such a threshold is challenged by factors such as the variability in insulin determination between different laboratories [31]. In this study, tertiles of QUICKI were used, with the lowest tertile depicting RIS, aligning with the methodology previously utilized, for instance, by Rajala et al. [13] and Vanhala et al. [32]. The cut-off value of QUICKI for RIS in this study (≤ 0.325) is reasonably in line with the cut-off values reported previously. Rajala et al. [13] reported a mean QUICKI value of 0.319 for individuals with DM and 0.334 for individuals with impaired glucose tolerance in comparison to a mean value of 0.335 for euglycemic individuals. Vanhala et al. [32], on the other hand, reported a QUICKI value of < 0.322 being predictive for type 2 DM in comparison to a QUICKI value of ≥ 0.340 (odds ratio 7.8) among individuals with body mass index ≥ 28 kg/m^2^. Hrebícek et al. [31] reported a QUICKI value < 0.357 being representative of IR. Despite thorough baseline assessment of metabolic status using fasting blood glucose and oral glucose tolerance test (OGTT) (and including only metabolically healthy participants), IS was measured only once during the follow-up. This represents a limitation, as temporal changes in IS could not be assessed.

Although the association of periodontal condition with RIS or IR in longitudinal study settings has been scarcely investigated, several cross-sectional studies exist. The findings of the present study are in line with a cross-sectional study by Gerin et al. [6] involving 63 participants aged 23–55 years, in which periodontitis was found to be associated with RIS, also after adjusting for body mass index. In addition, the findings of the present study were similar to those in a study by Kiryowa et al. [7] among 223 diabetic Ugandan participants aged ≥ 18 years, in which an association of periodontitis with IR was found. Moreover, in another large cross-sectional study by Demmer et al. [9] involving 3,616 diabetes-free participants, periodontitis was found to be associated with IR, with the finding being consistent across age, non-smoking, and non-obese subgroups. The findings of the present study also concur with the findings of a study involving 55 participants aged 32–57 years by Pulido-Moran et al. [8], in which periodontitis was associated with IR, with the notion that the Mediterranean diet did not appear to be a determining factor for IR in its association with periodontal disease. The finding of an increased risk of RIS among the edentulous participants in comparison to dentate participants with no deepened periodontal pockets is partly supported by the findings of a study on a Finnish population by Liljestrand et al. [33], who reported a slightly increased risk of DM among the edentulous participants compared to the dentate participants with 0–1 missing tooth.

Findings that conflict with the present study also exist. In a study by Islam et al. [10] involving 19,122 Korean participants aged ≥ 20 years, an association was observed between periodontitis and deregulation of beta cell function, but not with IR. Moreover, in another study involving 16,720 Korean participants aged ≥ 20 years [11], periodontitis was found to be associated with IR among postmenopausal women, but not among men or premenopausal women.

Evidence indicates that the inflammatory response in periodontitis extends beyond the oral cavity and has systemic effects [5, 34]. It has been proposed that inflammation in the periodontium contributes to the development of RIS and IR through three primary mechanisms. The first mechanism is the systemic microinflammation induced by periodontal inflammation, which downregulates insulin signaling via serine phosphorylation of insulin receptor substrate [35]. Secondly, bacterial products from periodontal tissues disseminate into the bloodstream, thereby diminishing the inhibitory effect of insulin on hepatic gluconeogenesis [36]. The role of periodontal bacteria is supported by the findings of a study by Demmer al. [37], who reported that microbial exposure via subgingival plaque containing Proteobacteria is associated with IR. The third proposed mechanism is gut dysbiosis caused by the ingestion of periodontal bacteria, leading to endotoxemia and changes in the blood metabolome [38]. However, the role of gut microbial carbohydrate metabolism in the development of IR is not yet fully understood [39].

An interesting finding in this study was the elevated risk estimates for RIS of the edentulous participants in comparison to the dentate participants without periodontal pockets; one could hypothesize that extraction of teeth would diminish the risk of systemic consequences. However, edentulous individuals often face difficulties with chewing, which can limit their intake of important sources of dietary fiber, such as fruits and vegetables [40], and adversely contribute to the management of IR [41]. Indeed, the proportion of edentulous participants with normal body composition was smaller, and an increase in WC during the follow-up period was more common among the edentulous than the dentate participants. In addition, the further exclusion of the participants with substantially increased risk of metabolic complications of the non-smoking participants and adjusting the analyses for confounders (including WC at follow-up and its change during the follow-up period) appeared to affect the risk estimates of the dentate participants with periodontal pockets (mainly elevating) more than the estimates of the edentulous participants (lowering). It is therefore plausible that a disturbed metabolic state, possibly related to unhealthy body composition, is such a strong risk factor for RIS that its confounding effect is difficult to handle completely by regression models. Unfortunately, no meaningful stratified analyses in each WC stratum could be performed due to the small size of this cohort data.

The effect of overall better health awareness on IS may be one possible explanation for the inconsistency of the observed association, evident especially in the above-mentioned subpopulation of the non-smoking participants without substantially increased risk of metabolic complications. There was, for instance, a clear discrepancy in the proportion of participants with increased risk of metabolic complications between the categories of the exposure variable in this subpopulation (35% of dentate participants without periodontal pockets, 36% of those with 1–6 sites with periodontal pockets, 20% of those with ≥ 7 sites with periodontal pockets, and 50% of the edentulous participants). In addition, the decrease in WC during the follow-up (between the follow-up examinations in 1996–1998 and 2007–2008) was greatest among those with ≥ 7 sites with periodontal pockets in this subpopulation. Furthermore, the number of teeth was greatest among these participants (Supplementary Table 1). It can be interpreted that poor periodontal condition represents a relatively weaker risk factor for RIS than the disturbed metabolic state related to an unhealthy body composition, and that poor periodontal condition may not be sufficient to counteract the ‘protective’ effect of a healthy body composition.

## Conclusions

Periodontal condition has been previously reported to be associated with the development of type 2 DM [42] and impaired glucose tolerance [43] in these Oulu1935 cohort data. While recognizing the limitations of the present study, the results provide further evidence of the link between periodontal condition and glycemic disorders.

Although further longitudinal studies with large study populations are needed, the results of the present study imply that maintaining good periodontal health is also beneficial in terms of normal insulin function. In light of the findings of this study, deteriorated oral health could be interpreted as an indicator for dentists to refer their patients for further medical evaluation by general medical practitioners and inform the patient about the links between oral health and general health.

## Supplementary Material



## Data Availability

The data supporting the findings were used under license and are not shared. Ville Myllymäki is the guarantor of this work and, as such, had full access to all the data in the study and takes responsibility for the integrity of the data and the accuracy of the data analysis.

## Geolocation information

Helsinki, Finland
