# Supplementary material for: Association of periodontal condition with insulin sensitivity – results of the Oulu1935 cohort study
Source: Acta Odontol Scand. 2026 Jul 16;85:46514. doi: 10.2340/aos.v85.46514 (PMC13379937; doi:10.2340/aos.v85.46514)
Supplement: Supplementary file 1 [file AOS-85-46514-s1.pdf]

Supplementary material has been published as submitted. It has not been copyedited or typeset by Acta Odontologica Scandinavica.

Supplemental material

Association of periodontal condition with insulin sensitivity—results of the Oulu1935 Cohort study

Ville Myllymäki[1,2], Pekka Ylöstalo[3,4,5], Anna Liisa Suominen[2,6,7], Matti Knuuttila[5], Ulla Rajala[5], Sirkka Keinänen-Kiukaanniemi[5,8,9], Sirpa Anttila[10], Tuomas Saxlin[2,6]

[1]Department of Oral and Maxillofacial Diseases, University of Helsinki and Helsinki University Hospital, Helsinki, Finland

[2]Institute of Dentistry, University of Eastern Finland, Kuopio, Finland

[3]Medical Research Center Oulu, Oulu University Hospital and University of Oulu, Oulu, Finland

[4]Department of Oral and Maxillofacial Surgery, Oulu University Hospital, Oulu, Finland

[5]Research Unit of Population Health, University of Oulu, Oulu, Finland

[6]Oral and Maxillofacial Diseases Teaching Unit, Wellbeing Services County of Northern Savo, Finland

[7]Welfare Epidemiology and Monitoring Unit, Finnish Institute for Health and Welfare, Helsinki, Finland

[8]Unit of Primary Health Care, Oulu University Hospital, Oulu, Finland

[9]Wellbeing Services County of North Ostrobothnia, Pyhäjärvi, Finland

[10]Dental Training Clinic, Oral Health Care, Wellbeing Services County of North Ostrobothnia, Finland

Running title: Periodontal condition and insulin sensitivity

Keywords: Epidemiology; Insulin resistance; Periodontal Diseases; Longitudinal Studies; Oral health

Correspondening author: Ville Myllymäki
ville.myllymaki@hus.fi

Supplemental material

Number of tables: 1 supplementary table

Supplementary Table 1. General characteristics of the non-smoking[†] participants without SIRMC[‡] (n = 145) according to the categories of periodontal condition at baseline in 1990–1992

	**Periodontal condition**				
	**Dentate without periodontal pockets (probing pocket depths [PPDs] < 4 mm)**	**Dentate with 1–6 sites with periodontal pockets (PPD ≥ 4 mm)**	**Dentate with ≥7 sites with periodontal pockets**	**Edentulous**	**Total**
Sex (baseline data), *n* (%)					
Men	20 (54)	17 (36)	12 (48)	8 (22)	57 (39)
Women	17 (46)	30 (64)	13 (52)	28 (78)	88 (61)
Number of teeth (baseline data), mean (SD)	15.4 (9.1)	18.1 (7.9)	20.5 (7.2)	-	17.7 (8.3)[§]
Reduced insulin sensitivity[¶] (follow-up data), *n* (%)					
Yes	6 (19)	15 (32)	3 (12)	10 (28)	34 (31)
No	31 (81)	32 (68)	22 (88)	26 (72)	111 (69)
Educational level (baseline data), *n* (%)					
Basic	29 (78)	35 (74)	15 (60)	33 (92)	112 (77)
Intermediate	4 (11)	5 (11)	7 (28)	2 (5)	18 (13)
High	4 (11)	7 (15)	3 (12)	1 (3)	15 (10)
Annual household income (baseline data), *n* (%)					
<€20,400	8 (22)	7 (15)	6 (24)	9 (25)	30 (21)
€20,400–30,599	8 (22)	15 (32)	2 (8)	13 (36)	38 (26)
€30,600–40,800	7 (19)	12 (26)	8 (32)	7 (19)	34 (23)
>€40,800	11 (29)	10 (21)	7 (28)	5 (14)	33 (23)
Missing data	3 (8)	3 (6)	2 (8)	2 (6)	10 (7)
Familial aggregation of diabetes mellitus (follow-up data), *n* (%)					
Low risk	18 (49)	26 (55)	17 (68)	20 (56)	81 (56)
Increased risk	19 (51)	21 (45)	8 (32)	16 (44)	64 (44)
Physical activity (baseline data), *n* (%)					
High physical activity	28 (76)	39 (83)	15 (60)	29 (80)	111 (77)
Low physical activity	9 (24)	6 (13)	10 (40)	6 (17)	31 (21)
Missing data	0 (0)	2 (4)	0 (0)	1 (3)	3 (2)
Food consumption habits (baseline data), *n* (%)					
Healthy diet	24 (65)	32 (68)	14 (56)	12 (33)	82 (57)
Moderately healthy diet	6 (16)	10 (21)	8 (32)	18 (50)	42 (29)

Unhealthy diet	4 (11)	3 (7)	2 (8)	6 (17)	15 (10)
Missing data	3 (8)	2 (4)	1 (4)	0	6 (4)
Smoking (follow-up data), *n* (%)					
Never smoker	21 (57)	27 (57)	10 (40)	16 (44)	74 (51)
Former smoker	16 (43)	20 (43)	15 (60)	20 (56)	71 (49)
Smoker	0	0	0	0	0
Waist circumference (WC [cm] [follow-up data]), mean (SD)	88.3 (6.9) (M) 78.1 (6.5) (W)	91.6 (6.0) (M) 77.4 (8.1) (W)	89.1 (5.5) (M) 78.8 (4.4) (W)	91.6 (7.2) (M) 80.5 (6.5) (W)	89.9 (6.4) (M) 78.7 (6.9) (W)
WC[††] (follow-up data), *n* (%)					
Normal	24 (65)	30 (64)	20 (80)	18 (50)	92 (63)
IRMC	13 (35)	17 (36)	5 (20)	18 (50)	53 (37)
WC change (cm) during the follow-up period[‡‡], mean (SD)	-1.8 (5.0)	0.1 (5.2)	-2.3 (6.3)	1.5 (5.7)	-0.4 (5.6)
WC change (cm) during the follow-up period[‡‡], *n* (%)					
WC change negative	24 (65)	25 (53)	17 (68)	14 (39)	80 (55)
0 ≤ WC change < 2	5 (13)	4 (9)	3 (12)	4 (11)	16 (11)
2 ≤ WC change < 4	4 (11)	8 (17)	2 (8)	4 (11)	18 (12)
WC change ≥4	4 (11)	10 (21)	3 (12)	14 (39)	31 (22)
Serum triglyceride level (follow-up data), mean (SD)	1.1 (0.4)	1.3 (0.7)	1.0 (0.4)	1.1 (0.4)	1.1 (0.5)
Serum high-density lipoprotein cholesterol level (follow-up data), mean (SD)	1.7 (0.5)	1.8 (0.5)	1.8 (0.4)	1.8 (0.5)	1.8 (0.5)
Arterial hypertension (follow-up data), *n* (%)					
No	15 (41)	25 (53)	9 (36)	15 (42)	64 (44)
Yes	13 (35)	16 (34)	13 (52)	13 (36)	55 (38)
Missing data	9 (24)	6 (13)	3 (12)	8 (22)	26 (18)

Abbreviation: SD, standard deviation.

[†]Current smokers excluded.

[‡]Participants with substantially increased risk of metabolic complications (SIRMC) excluded; waist circumference (WC) >102 cm for men and >88 cm for women on follow-up.

[§]Among dentate participants.

[¶]Defined based on the Quantitative Insulin Sensitivity Check Index (QUICKI) value in the lowest tertile of the study population (≤0.325). QUICKI was calculated according to the following equation: 1/[log(I0) + log(G0)], where I0 represents fasting insulin and G0 represents fasting glucose, both measured during the clinical examination.

[††]The cut-off points for WC: normal, ≤94 cm for men and ≤80 cm for women; and increased risk of metabolic complications (IRMC), >94–102 cm for men and >80–88 cm for women.

[‡‡]Between the follow-up examinations in 1996–1998 and 2007–2008.
